# Pharmacological interventions on early functional gastrointestinal disorders

**DOI:** 10.1186/s13052-016-0272-5

**Published:** 2016-07-16

**Authors:** Silvia Salvatore, Salvatore Barberi, Osvaldo Borrelli, Annamaria Castellazzi, Dora Di Mauro, Giuseppe Di Mauro, Mattia Doria, Ruggiero Francavilla, Massimo Landi, Alberto Martelli, Vito Leonardo Miniello, Giovanni Simeone, Elvira Verduci, Carmen Verga, Maria Assunta Zanetti, Annamaria Staiano, for the SIPPS Working Group on FGIDs

**Affiliations:** Department of Experimental and Clinical Medicine, Pediatrics, University of Insubria, Varese, Italy; Department of Pediatrics, Fatebenefratelli Hospital, Milan, Italy; Division of Neurogastroenterology and Motility, Department of Pediatric Gastroenterology, UCL Institute of Child Health and Great Ormond Street Hospital, London, UK; Department of Clinical, Surgical, Diagnostic and Pediatric Sciences, University of Pavia, Pavia, Italy; Department of Clinical and Experimental Medicine, Pediatric Unit, University of Parma, Parma, Italy; President Italian Society of Preventive and Social Pediatrics (SIPPS), Primary Care Pediatrician, Caserta, Italy; Primary Care Pediatrics, Milan, Italy; Interdisciplinary Department of Medicine-Pediatric Section, University of Bari, Bari, Italy; Department of Pediatrics, San Paolo Hospital, Bari, Italy; National Pediatric Healthcare System, Turin, Italy; Unit Research of Pediatric Pulmonology and Allergy Institute of Biomedicine and Molecular Immunology (IBIM), National Research Council, Palermo, Italy; Pediatric Department, Garbagnate Santa Corona Hospital, Milan, Italy; Department of Pediatrics, Aldo Moro University of Bari, Giovanni XXIII Hospital, Bari, Italy; Primary Care Pediatrics, ASL Brindisi, Mesagne, Italy; Department of Pediatrics, San Paolo Hospital, Department of Health Science, University of Milan, Milan, Italy; Primary Care Pediatrics, ASL Salerno, Vietri sul Mare, Italy; Department of Brain and Behavioral Sciences, University of Pavia, Pavia, Italy; Department of Translational Medical Sciences, Section of Pediatrics, Federico II University, Naples, Italy

**Keywords:** Regurgitation, Gastroesophageal reflux, Infant colic, Functional diarrhea, Dyschezia, Constipation, Cyclic vomiting syndrome, Treatment, Medication therapy management

## Abstract

**Background:**

Functional gastrointestinal disorders (FGIDs) are chronic or recurrent gastrointestinal symptoms without structural or biochemical abnormalities. FGIDs are multifactorial conditions with different pathophysiologic mechanisms including altered motility, visceral hyperalgesia, brain-gut disturbance, genetic, environmental and psychological factors.

Although in most cases gastrointestinal symptoms are transient and with spontaneous resolution in infancy multiple dietary changes and pharmacological therapy are often started despite a lack of evidence-based data. Our aim was to update and critically review the current literature to assess the effects and the clinical appropriateness of drug treatment in early (occurring in infants and toddlers) FGIDs.

**Methods:**

We systematically searched the Medline and GIMBE (Italian Group on Medicine Based on Evidence) databases, according to the methodology of the Critically Appraised Topics (CATs). We included reviews, clinical studies, and evidence-based guidelines reporting on pharmacological treatments. Systematic reviews and randomized controlled trials (RCTs) concerning pharmacologic therapies in children with early FGIDs were included, and data were extracted on participants, interventions, and outcomes.

**Results:**

We found no evidence-based guidelines or systematic reviews about the utility of pharmacological therapy in functional regurgitation, infant colic and functional diarrhea. In case of regurgitation associated with marked distress, some evidences support a short trial with alginate when other non pharmacological approach failed (stepped-care approach). In constipated infants younger than 6 months of age Lactulose is recommended, whilst in older ages Polyethylene glycol (PEG) represents the first-line therapy both for fecal disimpaction and maintenance therapy of constipation. Conversely, no evidence supports the use of laxatives for dyschezia. Furthermore, we found no RCTs regarding the pharmacological treatment of cyclic vomiting syndrome, but retrospective studies showed a high percentage of clinical response using cyproheptadine, propanolol and pizotifen.

**Conclusion:**

There is some evidence that a pharmacological intervention is necessary for rectal disimpaction in childhood constipation and that PEG is the first line therapy. In contrast, for the other early FGIDs there is a lack of well-designed high-quality RCTs and no evidence on the use of pharmacological therapy was found.

## Background

Functional gastrointestinal disorders (FGIDs) are defined as a variable combination of chronic or recurrent gastrointestinal symptoms not explained by structural or biochemical abnormalities [1].

To date, a complete understanding of the pathophysiology of FGIDs remains elusive. Physiological, intrapsychic, and sociocultural factors may amplify perception of infant discomfort by care-givers. Hence, the symptoms are often reported as severe, with impact on daily life activities and frequent use of different empiric treatments. It has been increasingly emphasized that FGIDs cannot be solved with the biological management, but need the biopsychosocial approach [2].

In the first few months of life FGIDs occur in up to 50 % of subjects with regurgitation and infantile colic representing the two most common conditions that usually spontaneously resolve or improve by 6 to 8 months of age. Other early (occurring in infants and toddlers) FGIDs include dyschezia, infant rumination syndrome, functional diarrhea, cyclic vomiting syndrome (CVS) and functional constipation (Table 1).

**Table 1:** Functional gastrointestinal disorders in infants and toddlers (according to Rome III classification)

Infant regurgitation	
Infant rumination syndrome	
Cyclic vomiting syndrome	
Infant colic	
Functional diarrhea	
Infant dyschezia	
Functional constipation	

Despite their favorable prognosis, such disorders are often extensively investigated and treated with multiple dietary changes and use of medications of uncertain benefit. Successful management is complicated by an incomplete pathophysiologic understanding of the disorders. Standard medical care consists of reassurance, education, and dietary advices [2]. If this approach is not effective, then pharmacological interventions are often prescribed.

Several drugs are used for treatment of FGIDs but no evidence-based therapy is available so far. Therefore, a systematic literature search was conducted on drug treatment of early FGIDs.

The aim of this article was to critically summarize the current evidences on the effects and the clinical appropriateness of pharmacological therapies in the treatment of FGIDs in preschool children.

## Methods

### Data sources and search strategy

We systematically searched the Medline and GIMBE databases using the following keywords: “gastric regurgitation”, “gastroesophageal reflux”, “cyclic vomiting syndrome”, “infant colic” “functional diarrhea”, “dyschezia”, “constipation”, “medication therapy management” or “treatment”, from January 2005 to June 2015, without any language restriction, limited to infant and preschool children. Additional strategies for identifying studies included the reference lists of review articles and included studies. The search has been based on a principle of hierarchical selection and has been conducted at least in double and in blind.

In the hierarchical selection, summaries of evidence, evidence-based guidelines (GL) and systematic reviews (SR) were searched primarily. The research was then completed according to theoretical saturation, with primary studies published after those included in the SR and those considered relevant entered as retrieved.

### Study selection

The target population of the interventions was represented by infant and toddlers with FGIDs, as defined by the Rome III criteria.

The possible outcomes considered in studies and SR were as follows: 1. The average duration and frequency (daily, weekly or monthly) of symptoms, or the reduction rate of the average number or duration of episodes 2. The reduction in the use of drugs; 3. The cost-effectiveness evaluation regarding number of pediatric visits, number of hospital accesses, changes of milk formulas, and the number of working days lost; 4. The duration of the child's sleep (min/day) whenever reported.

The intervention considered is the drug therapy, compared with non-pharmacologic therapy and/or no therapy.

For regurgitation we included the ESPGHAN-NASPGHAN [3] and NICE guidelines [4], a good quality systematic review [5] and a cohort study [6].

For cyclic vomiting syndrome we included the NASPGHAN 2008 Consensus Statement [7] and 3 reviews [8–10].

For infant colic we considered the 2013 Consensus [11] and a non-systematic review published in 2015 [12].

For functional diarrhea no systematic reviews or primary studies were found.

For infant dyschezia we consulted the ESPGHAN-NASPGHAN combined guidelines on constipation [13], a non-systematic review [11] and the Rome III Criteria document [1].

Finally, for constipation we considered the evidence-based 2014 ESPGHAN-NASPGHAN combined guidelines [13].

### Data analyses

The analysis and the evaluation of the evidences have been made in double, and in blind using checklists and validated criteria.

The analysis and evaluation of guidelines were carried out according to the following minimum criteria of validity: multidisciplinary panel, search for evidence, and grading of recommendations. SR analysis was performed using the AMSTAR (Assessment of Multiple Systematic Reviews) checklist [14].

For randomized studies, evaluation criteria for intervention studies of the Users’ Guides to the Medical Literature were used [15], while the analysis for other possible sources of bias was completed using the Cochrane Collaboration assessment of risk of bias validated instrument [16]. The studies of accuracy and diagnostic validation have been valued using the QUADAS 2 assessment tool [17].

## Results

Table 2 summarizes the results of pharmacological therapy in early FGIDs. There is a lack of evidences concerning drug treatment in infant rumination syndrome, functional diarrhea and infant dyschezia. The literature evidences and main indications for pharmacological treatment in the other early FGIDs are summarized below.

**Table 2:** Summary of evidences for Pharmacological Treatment of FGIDs

FGID	Results
Infant regurgitation	Alginate may be considered if symptoms are severe, associated with marked distress, and other non-pharmacological measures failed
Infant rumination syndrome	No evidence of pharmacological treatment
Cyclic vomiting syndrome	No RCTs. Retrospective studies showed benefit using cyproheptadine, propanolol and pizotifen in prophylaxis. During the acute phase of vomiting, intravenous infusion of glucoelctrolytic solutions, ondasetron, lorazepam and acid inhibitors are suggested.
Infant colic	No evidence of pharmacological treatment
Functional diarrhea	No evidence of pharmacological treatment
Infant dyschezia	No evidence of pharmacological treatment
Constipation	Lactulose is recommended for infants younger than 6 months of age, whereas in older infants and in children PEG with or without electrolytes represents the first-line therapy for both fecal disimpaction and maintenance.

### Infant regurgitation

Regurgitation is the passage of refluxed contents into the pharynx or mouth. In young infants, daily regurgitation is within the range of expected behaviors, and the great majority of infants are thriving and do not develop any disease [3]. Regurgitation reaches the peak at 4 to 6 months of age and decreases in frequency during the second semester of life [18]. Although regurgitation is a physiologic, frequent and spontaneously recovering phenomenon in healthy infants, almost 25 % of the parents are concerned and seek medical care [19].

Despite a wide use of drugs in infants with gastroesophageal reflux (GER) symptoms there are limited data on clinical outcome in the first year of life and there is no evidence that acid inhibitors or prokinetic agents improve regurgitation [20–22].

For H2-receptor antagonists only one small trial (35 participants) comparing famotidine to placebo reported results for infants and showed only a slightly reduced frequency and volume of regurgitation [23]. Eleven patients had no-serious, possibly drug-related adverse experiences including fussing or irritability, somnolence, anorexia, headache, vomiting, hiccups, and candidiasis. Guillet [24] first reported necrotizing enterocolitis in newborns treated with H2 blockers. Another cohort study [6] confirmed the increased risk in the newborns treated with ranitidine compared to controls (OR = 5.5).

Two double-blind, placebo-controlled trials in irritable infants with GER showed that both omeprazole and lansoprazole exert little effect on regurgitation and may be associated with adverse events [25, 26]. Another randomized single-blind study using esomeprazole for 8 days in 50 infants with GER symptoms did not report a significant clinical improvement [27]. The FDA reviewer experience published in 2012 [28] pointed out that in the 3 double-blinded and placebo-controlled clinical trials testing esomeprazole, lansoprazole, and pantoprazole, there were no statistically significant between-group differences.

The ESPGHAN-NASPGHAN guidelines do not recommend any pharmacological treatment in regurgitation [3]. Acid inhibitors are restricted to GER-disease demonstrated by investigations reserved to infants with alarm symptoms [3, 5].

The NICE guidelines in 2015 do not indicate a routine treatment in infants with regurgitation without marked distress or “red flags” symptoms or signs. However, a short trial with alginate (for 1–2 weeks) is considered when regurgitation is associated with marked distress and other non-pharmacological approach failed (stepped-care approach) [4].

A good quality systematic review [5] showed that the studies published so far on the efficacy of alginate in infants, present an underpowered population, and controversial results. A recent randomized controlled trial in 64 full-term infants with GER symptoms, showed a statistically significant improvement in overall symptoms evaluated through a validated questionnaire (I-GERQ) in the group treated with Mg alginate plus simethicone compared to the thickened formula or reassurance groups [29].

In conclusion there is no evidence that proton pump inhibitors (PPI), Histamine2 Receptor Antagonist or domperidone improves regurgitation whilst evidence of efficacy of alginate is still limited. Safety concerns are represented by neurological and cardiological effects for prokinetic, and by diarrhea/constipation, headache, hypergastrinemia and increased risk of infections for acid inhibitors.

### Cyclic vomiting syndrome

Cyclic vomiting syndrome (CVS) is a functional disorder characterized by recurrent (3 attacks in 6 months or 5 attacks in any time), individually stereotyped, transient (from a few hours up to 10 days) episodes of intense nausea and vomiting (at least 4 times/per hour for at least one hour) [30]. Attacks are separated by periods (one week or more) of complete well-being and no underlying pathology is recognised [30]. Despite the on-off pattern with inter-critical wellness, the severity and frequency of the acute emetic phases determine a negative impact of CVS on quality of life, frequent hospital referral, extensive investigations and, in some cases, a significant impairment of daily social activities and on school performance [31].

The incomplete knowledge of CVS pathophysiology currently limits the specificity and efficacy of its treatment. As reported in the NASPGHAN Guidelines [7] and in 3 more recent reviews [8–10] antimigraine, antiemetic, and anticonvulsant molecules have been studied in retrospective open-label trials for both prevention and acute phase with different and individual benefits. No prospective RCTs were identified.

As several precipitating (including psychological, physical, infective and dietary) factors are identified in about 2/3 of patients, a psychotherapeutic and nutritional support and avoidance of individual triggers should be considered before and in addition to pharmacological therapy.

Pharmacological prophylaxis is suggested if the crises are frequent (more than 6 episodes/year) and/or severe (requiring hospitalization) or disabling (lasting 3 days or longer). In children aged less than 5 years antihistamines such as cyproheptadine (0.25–0.5 mg/kg/day, twice per day) and pizotifen (0.5–1.5 mg/day, once or twice per day) are considered as “first-line therapy”. Propranolol (0.25–1 mg/kg/day in 2–3 administrations) is suggested as “second-line drug”, but is contraindicated in children with asthma, diabetes, depression and heart disease. Heart rate monitoring during the first days of therapy is recommended in all patients as well as discontinuation: tapering for 1–2 weeks. When the diagnosis for CVS is confirmed and individualized therapy is initiated, low dose first line drug should be progressively increased every 1 to 4 weeks up to therapeutic range, for at least 2 cycles of vomiting episodes. If the medication is not effective in preventing the attack of episodes or if there are significant adverse effects, the second-line drug or combination therapy should be started.

Anti-emetic agents (ondansetron and granisetron), anti-anxiety agents (lorazepam), and anti-migraine agents (sumatriptan and zolmitriptan) have also been attempted in prodromal phase. 5HT1B/1D receptor agonists (sumatriptan) showed more efficacies in patients with family history of migraine. However, this drug is not approved for use in children. Tricyclic anti-depressive agent amitriptyline was considered in the NASPGHAN guidelines in 2008 more effective as prophylactic drug in children older than 5 years [7]. To minimize side effects, the treatment is commonly initiated at a single nighttime dose of 0.25–0.5 mg/kg/day and increased weekly by 5 to 10 mg until 1.0–1.5 mg/kg. The risk of ventricular arrhythmias is reduced by monitoring the QTc interval (to maintain it <460 msec) before and after reaching the targeted dose. Other side effects reported were constipation, sedation and behavioral changes [8–10].

The benefit of oral erythromycin (20 mg/kg/day) as a prokinetic agent, or anti-epileptic drugs such as valproic acid and phenobarbital reported in initial trials was not confirmed by other authors [8–10].

The incapacity of increasing the energy production required during stress conditions, as a consequence of mutations of mtDNA, predisposes patients to the beginning of a vomit cycle and perpetuates the dysfunction of autonomic nervous system neurons [32]. Retrospective studies suggest the use of mitochondrial supplements such as L-carnitine (50-100 mg per day divided into 2 or 3 doses to a maximum of 1 g three times a day) and coenzyme Q-10 (10 mg per kg per day in two or three divided doses up to a maximum of 100 mg three times a day). However, there is still insufficient data to recommend their use in all children to prevent or reduce the length and gravity of the cyclic vomit episodes [7–10].

During the acute phase of vomiting, intravenous infusion of glucoelctrolytic solutions (with 10 % dextrose to terminate ketosis) to prevent dehydration and electrolyte imbalance,antiemetic drugs (5-HT3 receptor antagonist Ondasetron at 0.3–0.4 mg/kg/dose every 6 h if necessary, with an upper limit of 20 mg/dose), sedatives, such as lorazepam (0.05–1 mg/kg per dose every 6 h) and acid inhibitors to manage epigastric pain and prevent esophageal damage are suggested [7, 33].

A quiet, dark room is a non-stimulating environment that will help patients sleep.

### Infant colic

Infantile colic was originally defined by Wessel as crying lasting three or more hours a day, at least 3 days a week and for at least 3 weeks [34]. According to Rome III Criteria infantile colics are defined as irritability paroxysms with fussiness or crying that starts and ends without clear reasons, lasting at least 3 h a day, 3 days a week, for at least one week, in healthy, well fed and thriving babies [2].

Numerous hypotheses have been generated and multiple etiologies have been proposed to explain infant colic, leading to a variety of possible treatments. There are no uniform criteria for a specific therapeutic approach and no medication has definitively proved to be beneficial.

There are neither evidence-based recommendations from GL nor dedicated SR.

The 2013 Consensus [11] and the review article by Vandenplas et al. in 2015 [12] included 6 studies evaluating: simethicone (anti-foaming agent), simethicone-lactase, cimetropium bromide (anticholinergic antimuscarinic antispasmodic molecule), dicycloverine (anti-cholinergic agent), trimebutine (weak opioid and antimuscarinic effects), and PPIs. None of the above treatment showed a significant effect and some of them, such as dicyclomine [35] and cimetropium, can cause potentially serious adverse reactions.

A recent SR including 5 randomized controlled trials (and 430 infants enrolled) did not support the use of PPIs to decrease infant crying and irritability and highlighted related side effects specifically increased risk of gastrointestinal and/or respiratory tract infections [36].

Two studies found a comparable decrease in crying duration in infants given simethicone or placebo [37, 38].

Drugs with antispasmodic activity are commonly used, based on the assumption that contractions of the intestinal smooth muscles cause colic. Dicyclomine was more effective than placebo in three trials [39–41], but dangerous side effects were described, such as respiratory symptoms, seizures, syncope, pulse rate fluctuations, muscular hypotonia, and even coma that contraindicate its use in infants [42].

Cimetropium bromide was reported to be more effective than placebo in reducing the duration of crying but lethargy, motion sickness and somnolence may occur [43].

### Constipation

The first step in managing functional constipation in infants is to educate and reassure the parents [13]. In infants younger than 6 months of age glycerine suppositories can be helpful if rectal emptying is necessary to provide acute relief whilst lactulose is recommended as maintenance therapy [12].

In infants older than 6 months, several studies evaluated the efficacy and safety of polyethylene glycol (PEG), both for fecal disimpaction and for maintenance therapy [44, 45]. PEG is an unabsorbable compound and is not digested by colonic bacteria. Its mechanism of action is increasing osmotic load in the large intestine, which results in expansion of stool volume [46].

Successful disimpaction occurred in 75–92 % of children after 3–6 consecutive days, with the most effective dose being 1.0–1.5 g/kg per day [46]. A RCT showed that the use of oral PEG solution (1–1.5 gr/kg/die) for 6 consecutive days has the same efficacy of retrograde enemas. Bekkali et al demonstrated that successful disimpaction was achieved in 80 % of the children using retrograde enemas once daily and in 68 % of children using oral PEG solution for 6 consecutive days [47].

PEG with or without electrolytes is safe and its collateral effects, as diarrhea and abdominal pain, are determined by its mechanism of action. In a prospective observational study, Pashankar et al failed to find any side effects following PEG therapy [48].

Once the impaction has been removed, the treatment focuses on the prevention of recurrence. The main pharmacological agents are osmotic and stimulant laxatives. Good quality clinical trials are lacking on effectiveness of stimulant laxatives as maintenance therapy of childhood constipation.

Lactulose is an unabsorbable, osmotically active carbohydrate, which drags water into the gut, keeps the stools soft and facilitates their passage avoiding pain. Two randomized controlled trials comparing lactitol and lactulose found that both are equally effective in increasing stool frequency and normalizing stool consistency [49–51].

Candy et al., in a randomized, double-blind study, noted that PEG with electrolytes was more effective compared to lactulose in increasing defecation frequency in children with intractable constipation [45].

Loening-Baucke, comparing PEG without electrolytes with milk of magnesia, showed that no difference was found [52]. In another randomized study, PEG and milk of magnesia were equally effective in the long-term treatment of children with constipation and fecal incontinence [53].

Evidence does not support the use of mineral oil, which could lead to a risk of lipoid pneumonia, due to lung aspiration.

The 2014 ESPGHAN-NASPGHAN combined guidelines state that PEG and enemas are equally effective for fecal disimpaction. PEG administered orally is associated with a higher frequency of fecal incontinence during treatment of the fecal impaction compared with enema; however, based on the argument that PEG can be administered orally, the working group decided that PEG is to be preferred (1.1.5 g/kg/die) [13]. The use of PEG with or without electrolytes is recommended as first-line maintenance treatment and the starting dose is 0.4 g/kg/day. PEG resulted better than lactulose, milk of magnesia and mineral oil [13]. The use of enemas for maintenance therapy is not recommended in children with constipation. Glycerin suppositories can be considered for occasional use in disimpaction [13].

## Discussion

Functional symptoms during childhood may be related to normal development (e.g., infant regurgitation), or to a maladaptive behavioral response to internal or external stimuli (e.g., in functional constipation, retention of feces can be considered as a “learned response” to painful defecation or to forced toilet training).

Physiological, intrapsychic, and sociocultural factors amplify the perception of these symptoms so they may be experienced as severe, troublesome, or threatening, with subsequent impact on family quality of life, daily activities and medical care [54].

Over the last few years a great bulk of evidence has shown the crucial role of the so-called brain-gut axis in the pathophysiology of gastrointestinal diseases [55]. Through this bidirectional communication system, signals from the brain can modulate various intestinal functions (motility, secretion, blood flow, and gut-associated immunity) and conversely, visceral messages from the gastrointestinal tract can influence brain activity. Bidirectional signals between the gut and the brain are regulated at neural, immunological and hormonal levels, and involve the hypothalamic-pituitary-adrenal axis and the enteric microbiota. The neural pathways include the central nervous system, the sympathetic and parasympathetic arms of the autonomic nervous system and the enteric nervous system [56, 57]. Alteration of the neural-gut cross-talk and network may be involved in the pathophysiology of functional and inflammatory gastrointestinal disorders.

Despite recent advances in pathophysiology, there is still no biological specific marker of FGIDs. Therefore, the diagnosis is currently clinically based from a set of symptoms with exclusion of warning signs. It is also known that FGIDs cannot be solved with the biological management, but need the biopsychosocial approach. According to this model, symptoms are both physiologically multi-determined and modifiable by sociocultural and psychosocial influences.

An improved awareness and knowledge of FGIDs are essential to help general practitioners and pediatricians in the pharmacological and non pharmacological management of these frequent conditions.

Infants presenting with gastrointestinal problems such as regurgitation, cyclic vomiting syndrome, infantile colic and defecation problems often undergo a series of unnecessary investigations and medical treatments [2].

In the last 2 decades, PPI prescriptions dramatically increased in pediatric patients. As the use of acid-suppressive medication has risen, many have questioned the effectiveness and safety of these agents [58, 59]. Although suppression is effective in the treatment of rare acid-related symptoms or erosive esophagitis in children, RCTs and SR have demonstrated a lack of efficacy of PPIs, specifically in treating regurgitation or other GER symptoms in infants [60]. In regurgitation treatment, limited data and the NICE guidelines support a short trial with alginate if symptoms are severe, troublesome and non responsive to behavioral and dietetic modifications.

In functional constipation, lactulose is recommended for infants younger than 6 months of age, whereas PEG has been shown to be more effective in older children. Particularly, when compared to other laxatives, PEG is more active in increasing stool frequency and decreasing difficulty in passing stools and thus represents the first-line therapy for both fecal disimpaction and maintenance. Conversely, no evidence supports the use of laxatives for infant dyschezia.

We found no evidence-based GL or SR about the utility of pharmacological therapy in infantile colic, rumination and functional diarrhea. Moreover, no RCTs regarding the pharmacological treatment of CVS are available, but retrospective studies showed a good (individual) clinical response to drugs such as cyproheptadine, propanolol and pizotifen in prohylaxis in young children. During the acute phase of vomiting, intravenous infusion of glucoelctrolytic solutions, ondasetron, lorazepam and acid inhibitors are suggested.

## Conclusion

Evidence supporting a pharmacological approach to early FGIDs is scarce except than in constipation, thus justifying caution when prescribing drugs to these patients. Further research is needed to clarify the underlying mechanisms of these disorders and the efficacy of pharmacological treatments in early FGIDs.

## Abbreviations

AMSTAR, Assessment of Multiple Systematic Reviews; CATs, Critically Appraised Topics; CVS, cyclic vomiting syndrome; FGIDs, Functional gastrointestinal disorders; GER, gastroesophageal reflux; GIMBE, Italian Group on Medicine Based on Evidence; GL, evidence-based guidelines; PEG, Polyethylene glycol; PPI, proton pump inhibitors; RCT, randomized controlled trial; SR, systematic reviews
